# Aneuploid serves as a prognostic marker and favors immunosuppressive microenvironment in ovarian cancer

**DOI:** 10.1186/s13048-024-01356-w

**Published:** 2024-02-02

**Authors:** Ming Du, Qingqing Cai, Jiaan Sun, Mingxing Zhang, Shuo Zhang, Xiaoxia Liu, Mengyu Zhang, Xiaoyan Zhang

**Affiliations:** 1 Obstetrics and Gynecology Hospital, Fudan University, Shanghai, 200011 China; 2 Shanghai Key Laboratory of Female Reproductive Endocrine Related Diseases, Shanghai, 200011 China; 3 Department of Obstetrics and Gynecology of Shanghai Medical School, Fudan University, Shanghai, 200032 China; 4 Center for Reproductive Medicine, Naval Medical Center, Naval Medical University, Shanghai, 200052 China

**Keywords:** Ovarian cancer, Aneuploid, Chromosome alteration, Immune microenvironment, Antigen presentation

## Abstract

Ovarian cancer is the most lethal gynecologic neoplasm, and most patients experience recurrence and chemoresistance. Even the promising immunotherapy showed limited efficacy in ovarian cancer, probably due to the immunosuppressive microenvironment. However, the behind mechanisms of the immune exclusion or cold phenotype in ovarian cancer still remain to be explored. As a cancer dominated by copy number variations instead of mutations, ovarian cancer contains a high fraction of aneuploid, which might correlate with immune inhibition. Nevertheless, whether or how aneuploid affects ovarian cancer is still unclear. For exploring the role of aneuploid cancer cells and the potential ploidy-immune relationship, herein, the ploidy information was first comprehensively analyzed combining the karyotype data and copy number variation data obtained from Mitelman and cBioPortal databases, respectively. Ovarian cancer showed strong ploidy heterogeneity, with high fraction of aneuploid and recurrent arm-level and whole chromosome changes. Furthermore, clinical parameters were compared between the highly-aneuploid and the near-diploid ovarian cancers. Aneuploid indicated high grade, poor overall survival and poor disease-free survival in ovarian cancer. To understand the biofunction affected by aneuploid, the differentially expressed genes between the highly-aneuploid and the near-diploid groups were analyzed. Transcription data suggested that aneuploid cancer correlated with deregulated MHC expression, abnormal antigen presentation, and less infiltration of macrophages and activated T cells and higher level of T cell exclusion. Furthermore, the ploidy-MHC association was verified using the Human Protein Atlas database. All these data supported that aneuploid might be promising for cancer management and immune surveillance in ovarian cancer.

## Introduction

Ovarian cancer is the most lethal gynecological neoplasm, with less than 30% five-year survival [1]. More than 80% patients experience recurrence or chemotherapy-resistance and show unsatisfying response to the second-line chemotherapy [2]. The immune therapy has been promising in ovarian cancer since the pembrolizumab was approved for the treatment of solid tumors with high microsatellite instability and mismatch repair defects [3]. However, up to now, the PD-1/PD-L1 immune checkpoint blockade (ICB) therapy still shows limited curative efficacy, with less than 20% objective response rate in ovarian cancer [4].

The frustrating response to immune therapy might be attributed to the immunosuppressive microenvironment in ovarian cancer. Compared with other solid tumors, ovarian cancer has less tumor mutation burden (TMB) and less immunogenicity [5, 6]. The process of antigen processing and presentation is aberrant in ovarian cancer, with dysfunction of antigen-presenting cells and deregulated expression of MHC-related molecules [7, 8]. As a “cold” type cancer, ovarian cancer, especially platinum-resistant subtype, shows low infiltration of cytotoxic immune cells, such as CD4 + T cell and CD8 + T cell [9, 10]. Uncovering the contributing factors of immunosuppressive microenvironment might be beneficial for immune reactivation and efficacy improvement in ovarian cancer.

Aneuploid, one prominent type of genomic features in ovarian cancer, might provide some inspirations for the research of immunosuppressive microenvironment. Pan-cancer copy number variation (CNV) data showed that ovarian cancer was manifested as aneuploid and high ploidy value [11, 12]. Recent CNV data from low-pass whole genome sequencing suggested that ovarian cancer presented strong heterogeneity of ploidy status, and aneuploid indicated advanced stage and poor survival [13]. As a neoplasm driven by CNV, ovarian cancer was categorized into the “C” type cancer, instead of the “M” cancer driven by mutation, with low level of TMB and low occurrence of neoantigens [14, 15]. Previous pan-cancer data also found the potential correlation between aneuploid and immune evasion or less leukocyte infiltration [16, 17]. Overall, current ploidy studies partially support that aneuploid might participate in the immunosuppressive microenvironment, although the ploidy-immune relationship still remains to be elucidated.

For better understanding the role of aneuploid in the immune microenvironment in ovarian cancer, herein, we analyzed the ploidy status of ovarian cancer and preliminary explored the ploidy-immune relationship in ovarian cancer. This research might provide information for ploidy research in ovarian cancer and supplement new mechanisms for the immunosuppressive microenvironment formation.

## Materials and Methods

### Patient cohort

For karyotype analysis, 455 patients with ovarian adenocarcinoma from Mitelman Database (https://mitelmandatabase.isb-cgc.org/) were included in this study. For aneuploid status analysis, 552 patients with ovarian serous cancer from the Cancer Genome Atlas (TCGA) were included in this study.

### Ploidy analysis

Karyotype landscape of ovarian cancer was analyzed among 455 patients. The karyotype data and recurrent specific chromosome changes were downloaded from Mitelman Database. The karyotypic analysis was based on the International System for Human Cytogenetic Nomenclature (ISCN) [18]. According to ISCN, cells with a chromosome modal number between 35 and 45 are hypodiploid and between 47 and 57 hyperdiploid; cells with a modal number between 58 and 68 are hypotriploid and between 70 and 80 hypertriploid; cells with a modal number between 81 and 91 are hypotetraploid and between 93 and 103 hypertetraploid. More than one type ploidy contained in one patient were considered as a chimeric ploidy status. As for recurrent specific chromosome change, gains and losses are assessed at the corresponding ploidy level.

Ploidy status of ovarian cancer was analyzed among 552 patients. The aneuploidy score (AS, the score for estimating the cancer ploidy status and aneuploid degree) obtained from the CNV analysis using the ABSOLUTE algorithm (an algorithm assessing the CNV data, purity value and ploidy value of cancer using R package), can partially reflect the ploidy status of cell lines and tumor tissues [11, 16, 19]. AS was downloaded from cBioPortal (www.cbioportal.org). Patients were ranked by aneuploid score and the classification was performed using the quartile method according to previous cell line analysis [19]. The top quartile was categorized into the “highly-aneuploid” or the “high AS” group with high aneuploid score, while the bottom quartile into the “near-diploid” or the “low AS” group with low aneuploid score.

### Survival and clinicopathologic characteristics analysis

The survival data of 552 patients was downloaded from TCGA. The survival R package was used for survival analysis, including overall survival (OS), disease specific survival (DSS), disease free survival (DFS), and progression free survival (PFS). The clinicopathologic characteristics were downloaded from TCGA, including the International Federation of Gynecology and Obstetrics (FIGO) stage, tumor grade, age, metastasis.

### Identification of DEGs and enrichment analysis

Comparison analysis of transcription data was preliminarily performed using cBioPortal online tool between the highly-aneuploid versus the near-diploid. Limma R package was used for identification and analysis of the differentially expressed genes (DEGs) between the two groups. DEGs were determined by a log_2_FC of 1 and an adjusted *P* value of 0.05. Gene Ontology (GO) analysis and Kyoto Encyclopedia of Genes and Genomes (KEGG) pathway analysis of DEGs were performed using Metascape analysis tool [20]. Gene Set Enrichment Analysis (GSEA) was also performed. For GSEA analysis, the thresholds for enrichment results were set to q = 0.05 and *p*-value = 0.05.

### Immune-related analysis

The immune infiltration of the two groups was analyzed using the single sample Gene Set Enrichment Analysis (ssGSEA) algorithm. The TMB (the number or density of non-synonymous mutations on the coding region) of the two groups was analyzed for indirectly accessing the probability of neoantigens. For estimating the overall immune infiltration and function, the Estimate Score (the score to estimate the immune microenvironment, which generated from the Immune Score (the score assessing the immune cell infiltration in tumor) and the Stromal Score (the score assessing the stromal cell infiltration)) of the two groups was analyzed using the Estimate algorithm [21]. For estimating the immune evasion, especially the dysfunction and the exclusion of T cells, the Tumor Immune Dysfunction and Exclusion (TIDE) score (the score applied to assess the immune evasion and the response to immunotherapy, including the Exclusion Score (assessing the T cell exclusion situation) and the Dysfunction score (assessing the T cell function)) of the two groups was analyzed using TIDE database (http://tide.dfci.harvard.edu/). The expression level of 60 immune checkpoint genes was analyzed for the immunotherapy efficacy prediction [22]. For protein verification of MHC assembly and the markers of immune cells and immune checkpoints, the Human Protein Atlas database (HPA) (https://www.proteinatlas.org/) was used for the correlation analysis between aneuploid and immune function.

### Statistical analysis

Difference of AS, ploidy value, age of 552 patients were analyzed with the Wilcoxon test. The FIGO stage and tumor grade were analyzed using the Chi-squared test. The protein level between the aneuploid and the near-diploid was analyzed using the unpaired t test. *P* < 0.05 was considered statistically significant.

## Results

### Ploidy analysis of ovarian cancer patients from Mitelman and CbioPortal database

**Fig. 1:**
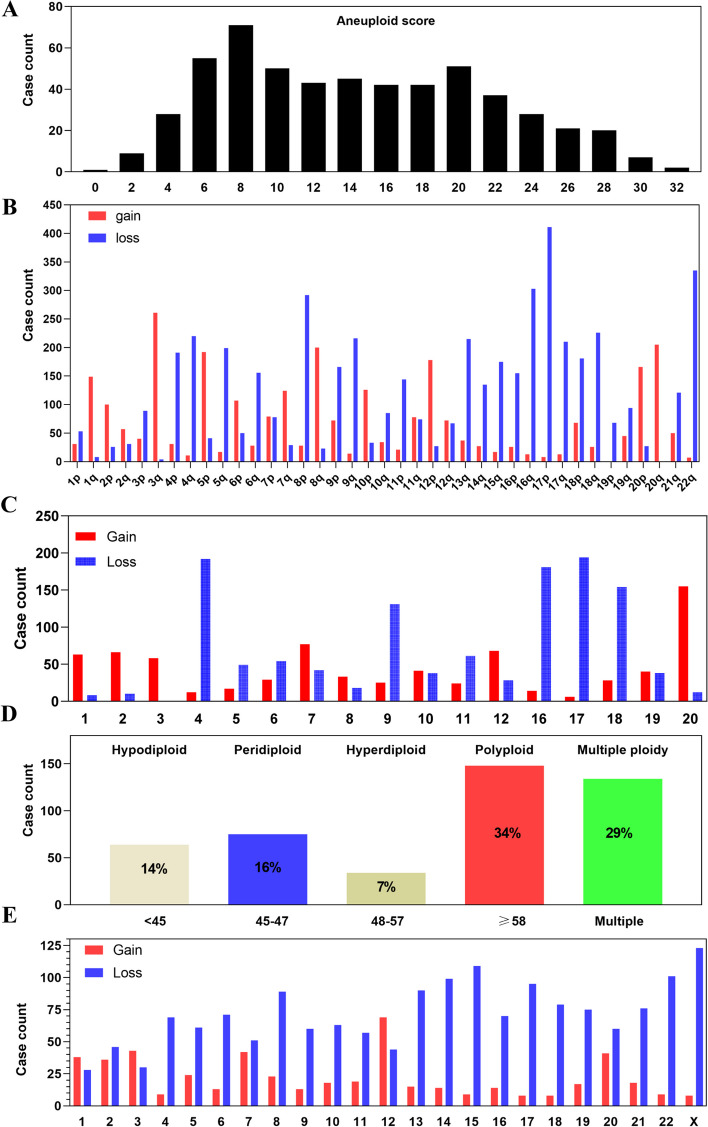
Aneuploid score and karyotype information of ovarian cancer. **A** Aneuploid score of 552 ovarian cancer patients. The aneuploid score is obtained from cBioPortal using CNV data and ABSOLUTE algorithm. **B** Arm-level CNV of ovarian cancer (Blue = arm-level deletion; Red = arm-level amplification). **C** Whole chromosome-level CNV of ovarian cancer (Blue = chromosome loss; Red = chromosome gain). **D** Karyotype landscape of ovarian cancer. Terms from left to right are hypodiploid (chromosome number < 45), peridiploid (chromosome number between 45 to 47), hyperdiploid (chromosome number between 48 to 57), polyploid (chromosome number ≥ 58), Multiple ploidy (more than one ploidy status), respectively. **E** Recurrent chromosome changes in ovarian cancer (Blue = chromosome loss; Red = chromosome gain)

The aneuploidy score of 552 ovarian cancer patients was obtained via cBioPortal online analysis, which is based on CNV data and ploidy estimation using the ABSOLUTE algorithm. Most ovarian cancer cell lines were aneuploid. Most ovarian cancers had relatively high aneuploid scores (Fig. 1A). Analysis of arm-level chromosome changes showed that the deletions of 8p, 16q, 17p and 22q, the amplifications of 3q, 8q,12p and 20q were more common arm-level chromosome alterations (Fig. 1B).

**Fig. 2:**
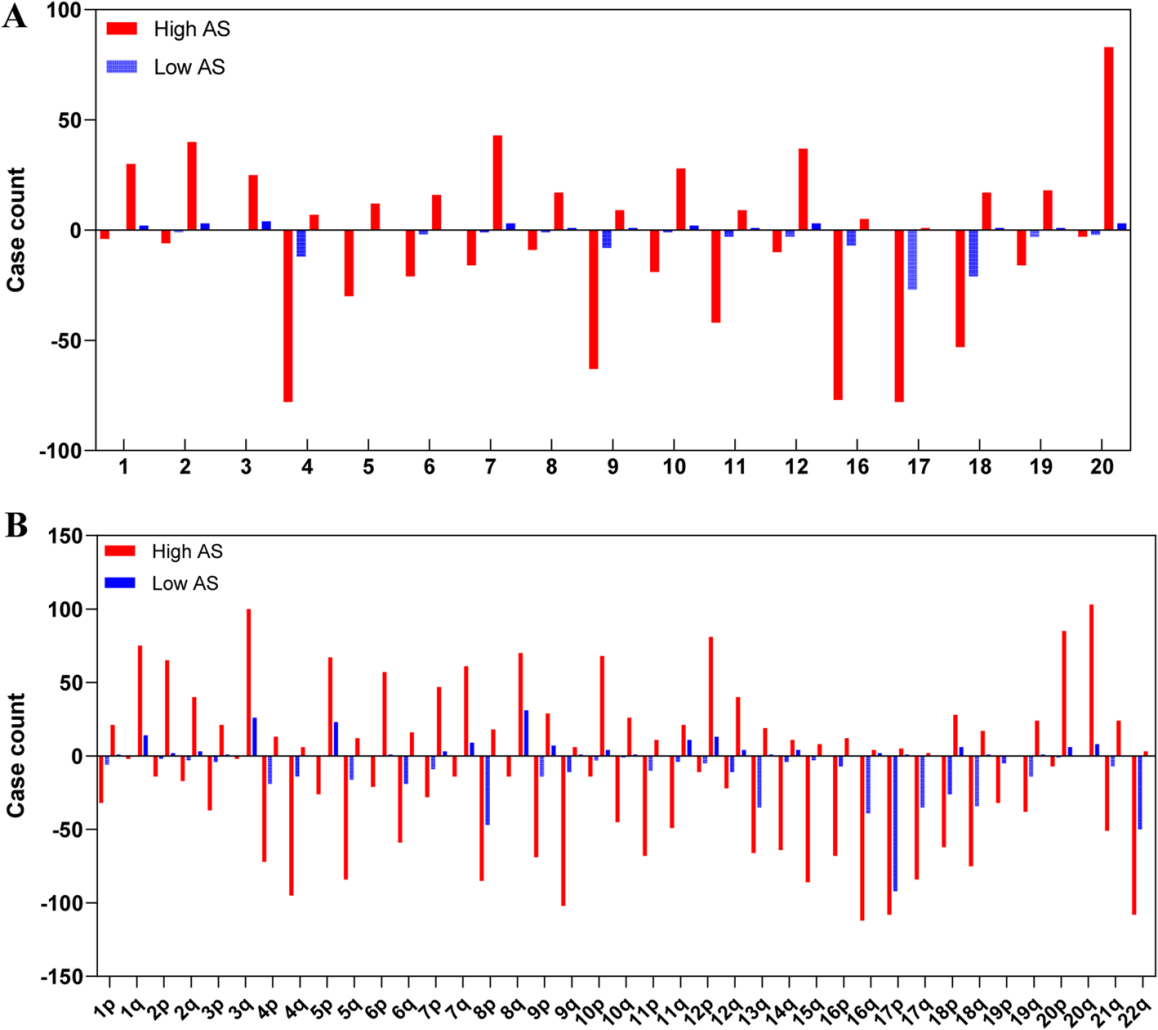
Comparison analysis of arm-level and whole chromosome changes between the highly-aneuploid and the near-diploid groups. (Red = the highly-aneuploid group with high aneuploid score; Blue = the near-diploid group with low aneuploid score)

The karyotype data of 455 ovarian cancer patients was obtained from Mitelman database. According to the karyotype analysis, ovarian cancer showed strong karyotype heterogeneity and most ovarian cancers were aneuploid (Fig. 1D). Only 16% patients maintained the diploid karyotype, while most patients contained complex karyotypes. Approximately 30% ovarian cancers presented more than one ploidy status. Both the CNV data and karyotype data showed the losses of chromosome 8 (chr 8), chr 15, chr 22, chr 19 and chr X, the gains of chr 12, chr 20 were more common chromosome alterations (Fig. 1C and E). Comparison analysis of arm-level or whole chromosome alterations showed that chromosome changes in ovarian cancer might be mainly contributed by the aneuploid subtype (Fig. 2).

### Clinical value of aneuploid in ovarian cancer

**Fig. 3:**
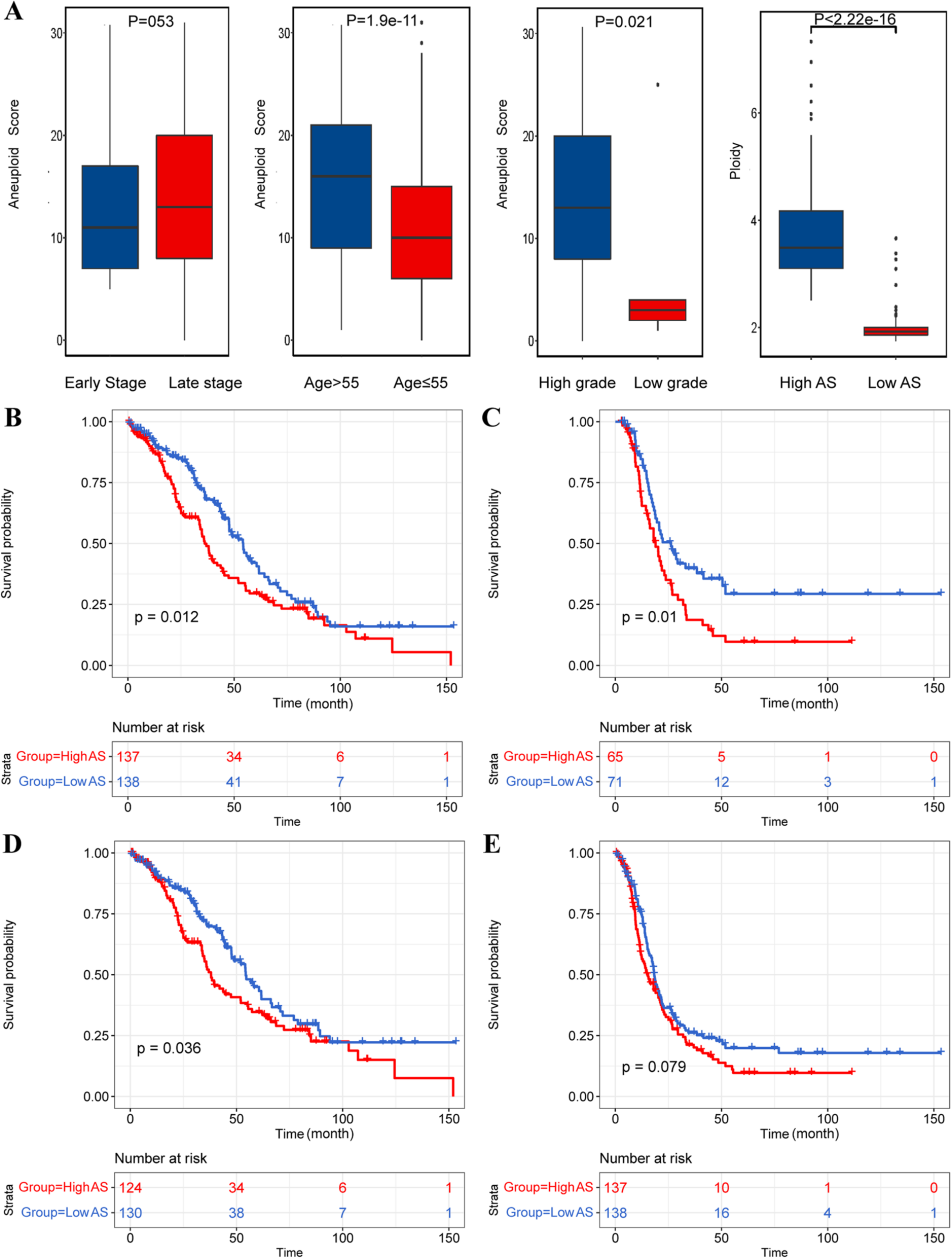
Correlation analysis between clinicopathologic parameters and aneuploid score. A AS comparison among patients with different clinicopathologic features. From left to right: early- and late-stage ovarian cancer (early stage = stage I and stage II, *p* > 0.05), age (cutoff = 55, *P* < 0.05), grade (low grade = grade 1, *P* > 0.05), and ploidy value(*P* < 0.05). B Comparison of overall survival between the aneuploid and the near-diploid groups (Red = high AS, Blue = low AS; *p* < 0.05). C**:** Comparison of disease-free survival between the highly-aneuploid and the near-diploid groups (*p* < 0.05). D Comparison of disease specific survival between the highly-aneuploid and the near-diploid groups (*p* < 0.05). **E** Comparison of progression-free survival between the highly-aneuploid and the near-diploid groups (*p* > 0.05)

For demonstrating the potential clinical value of aneuploid in ovarian cancer, comparison analysis of multiple clinicopathologic parameters was performed. It was suggested that there was no significant difference of aneuploidy score between early and advanced stage, although the AS of the late stage was slightly higher than the early stage (Fig. 3A). Patients over 55 years old showed significantly higher AS than those under the age of 55 (Fig. 3A). High grade ovarian cancers (Grade 3 and Grade 4) had higher AS than low grade (Fig. 3A). The ploidy value of the highly-aneuploid or the high AS group (Mean = 3.77) was significantly higher than the near-diploid group or the low AS group (Mean = 1.97) (Fig. 3A). High AS indicated worse overall survival, poor disease-free survival and poor disease specific survival than the low AS group (Fig. 3B-E).

### Difference in expression profiles between the near-diploid and the aneuploid ovarian cancer patients

**Fig. 4:**
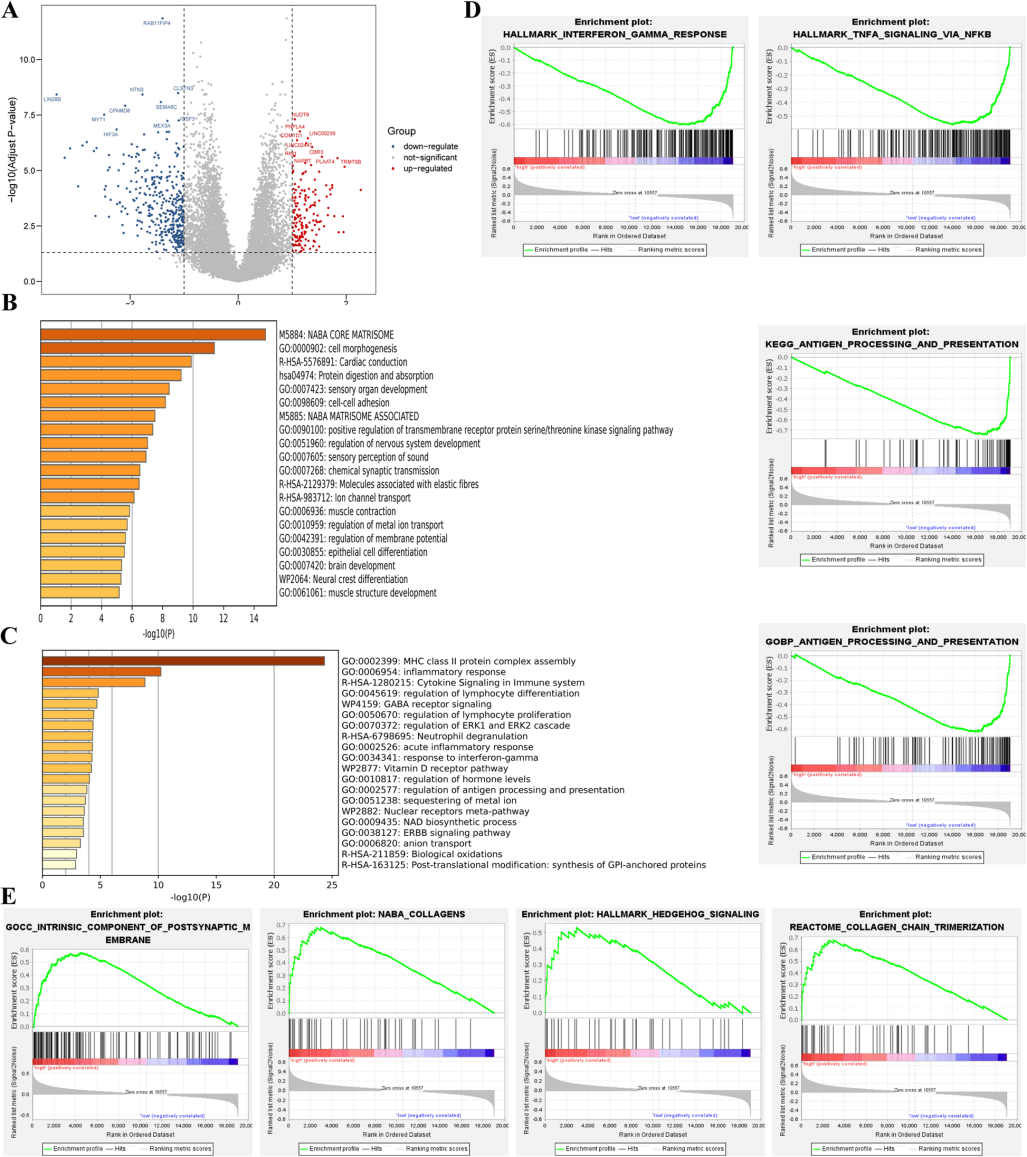
Identification and functional analysis of the differentially expressed genes between the highly-aneuploid and the near-diploid groups. **A** The volcanic plot of the DEGs between high AS versus. low AS. **B** Enrichment analysis of the upregulated genes in high AS group.** C** Enrichment analysis of the the downregulated genes in high AS group.** D** Significant gene sets from the GSEA of the downregulated genes in high AS group. **E** Significant gene sets from the GSEA of the upregulated genes in highly-aneuploid group

To better understand the contribution of aneuploid in cancer initiation and progression, the DEGs were identified and analyzed between the high AS group and the low AS groups. Compared with the near-diploid ovarian cancer, 411 genes were downregulated and 167 genes were upregulated in the aneuploid group (Fig. 4A). To understand the potential bioprocess or biofunction of these DEGs, GO analysis and KEGG analysis were carried out using the Metascape analysis tool. According to the enrichment analysis, genes upregulated in the aneuploid group were mainly involved in pathways in “NABA core matrisome”, “Cell morphogenesis”, “Protein digestion and absorption”, and “positive regulation of transmembrane receptor protein serine kinase signaling pathway” (Fig. 4B). Genes downregulated in aneuploid ovarian cancer were particularly enriched in the immune-related bioprocesses, such as “MHC class II protein complex assembly”, “Inflammatory response”, “Cytokine signaling in immune system”, “Regulation of lymphocyte differentiation” and “Regulation of lymphocyte proliferation” (Fig. 4C).

**Fig. 5:**
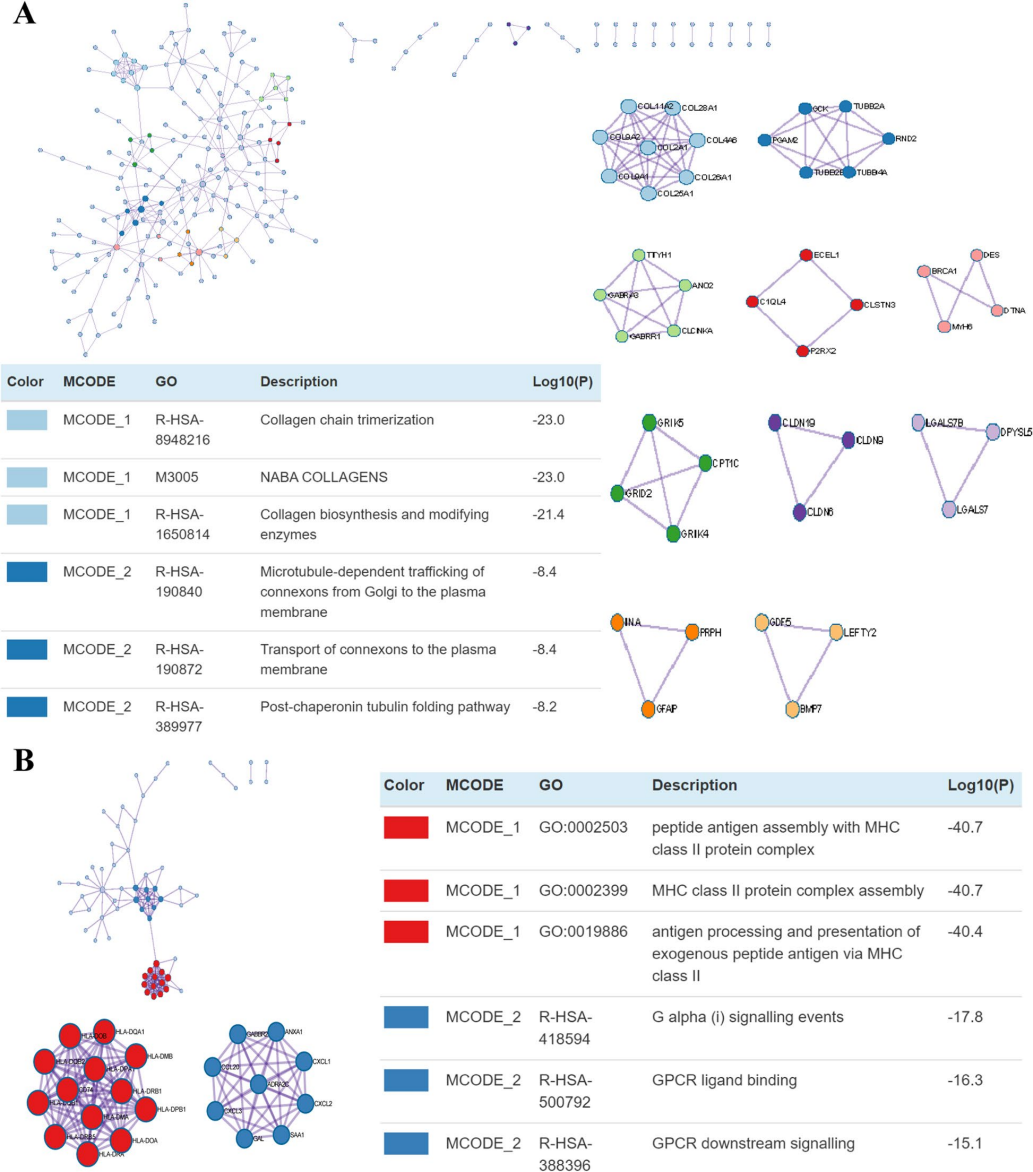
Protein–protein Interaction Enrichment Analysis interactions. **A** PPI interaction of the up-regulated genes in the highly-aneuploid group. **B** PPI interaction of the down-regulated genes in the highly-aneuploid group

The GSEA analysis showed that genes upregulated in the aneuploid group were involved in the “Collagen chain trimerization”, “Collagens”, “Collagen biosynthesis and modifying enzymes”, “Intrinsic component of postsynaptic membrane”, “Intrinsic component of synaptic membrane”, “Neurotransmitter uptake” and so on (Fig. 4D). The genes downregulated in the aneuploid group were highly enriched in immune-related gene sets, especially “KEGG-antigen processing and presentation”, “GO-antigen processing and presentation of peptide antigen”, “GO-antigen processing and presentation of exogenous peptide”, “GO-MHC protein complex”, the gene sets related to the antigen presentation and “Hallmark-Interferon γ response”, “TNF-α signaling via NF-κB” (Fig. 4E). The Protein–protein Interaction analysis (PPI) showed that some interactions existed among the down-regulated and the up-regulated genes in the aneuploid ovarian cancer. For the up-regulated genes, interactions were enriched in collagen-related bioprocess and connexon-related bioprocess (Fig. 5A). For the down-regulated genes, interactions were enriched in MHC protein complex and antigen presentation (Fig. 5B).

### Relationship between aneuploid and abnormal antigen presentation

**Fig. 6:**
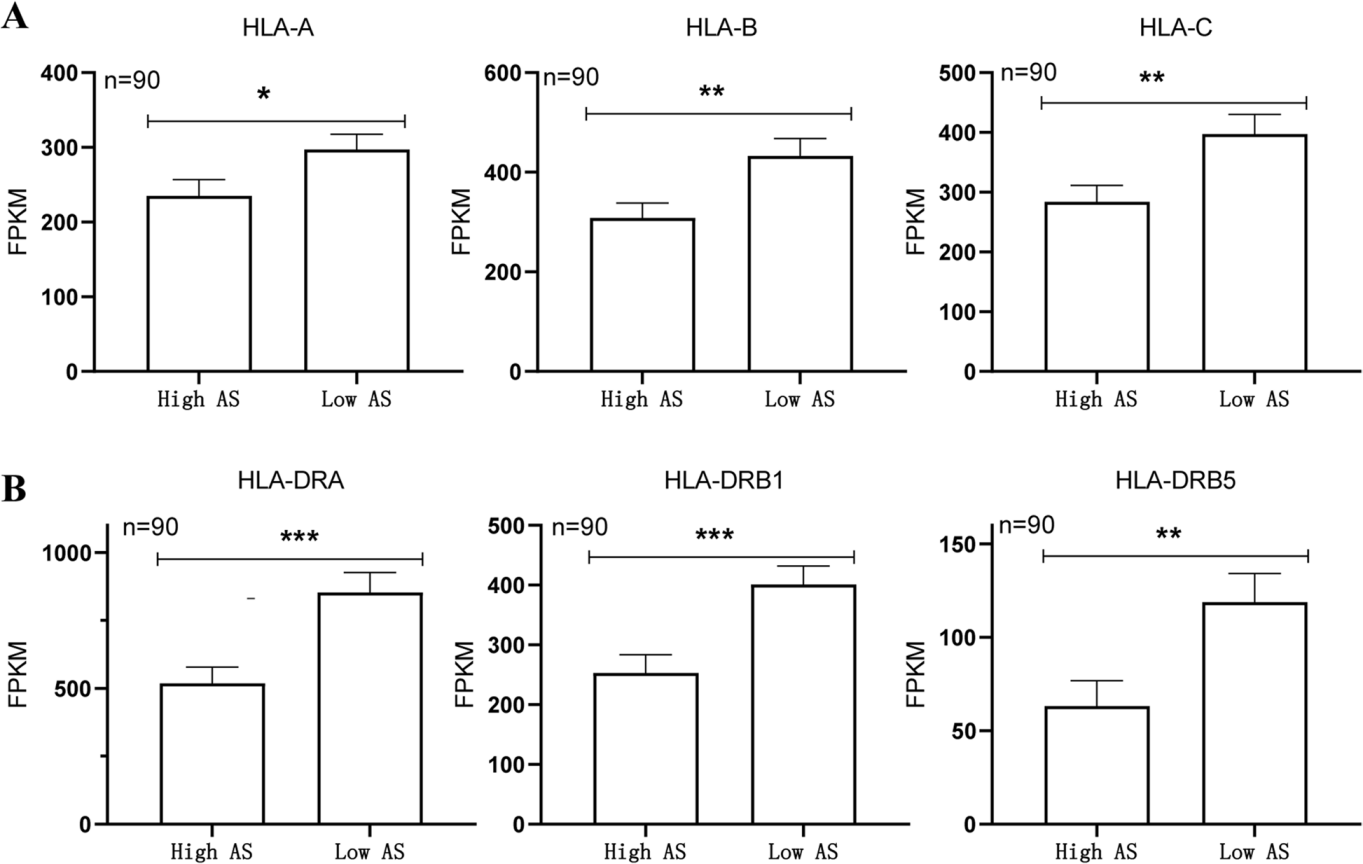
Comparison of MHC protein level between the highly-aneuploid and the near-diploid ovarian cancer patients. **A** Comparison of MHC I protein level between the highly-aneuploid and the near-diploid groups. **B** Comparison of MHC II protein level between the highly-aneuploid and the near-diploid groups. * *P* < 0.05, ** *P* < 0.01, *** *P* < 0.001

Transcription data showed the potential correlation between aneuploid and abnormal expression of genes of MHC protein and genes related to antigen presentation. For further verification of the ploidy-MHC relationship, HPA database was used for analysis of protein level of different MHC class protein members. A total of 357 ovarian cancer patients had both the AS score and the MHC protein data. According to the quartiles, 90 “highly-aneuploid” patients showed significantly lower protein level of MHC-I class member and MHC-II class member than 90 “near-diploid” patients, including HLA-A, HLA-B, HLA-C, HLA-DRA, HLA-DRB1, HLA-DRB5 (Fig. 6).

### Immune infiltration between the aneuploid and the near-diploid ovarian cancer patients

**Fig. 7:**
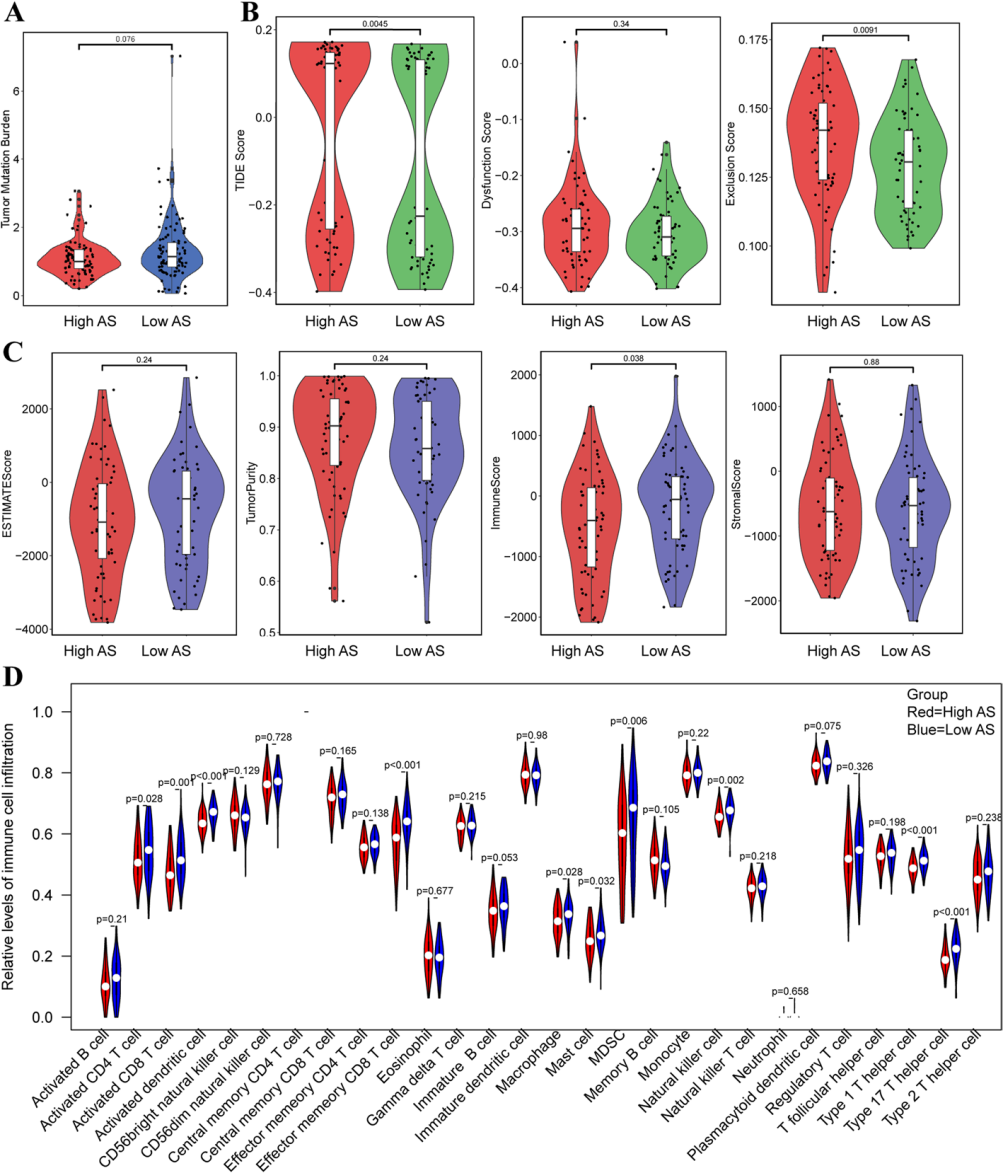
Comparison of immune function and infiltration between the highly-aneuploid and the near-diploid ovarian cancer patients. **A** Comparison of TMB between high AS and low AS (Red = high AS, Blue = low AS; *P* > 0.05).** B** Comparison of immune evasion situation between high AS and low AS (Red = high AS, Green = low AS). From left to right: TIDE score (*P* > 0.05), Exclusion score (*P* < 0.05), Dysfunction score (*p* > 0.05). **C** Comparison of estimate score between high AS and low AS (Red = high AS, Blue = low AS). From left to right: ESTIMATE score (*P* > 0.05), Tumor purity (*P* > 0.05), Immune score (*p* < 0.05) and Stromal score (*P* > 0.05). **D** Comparison of immune infiltration between high AS and low AS (Red = high AS, Blue = low AS)

The transcription data and HPA external validation preliminarily indicated the ploidy-immune association, which suggested that the aneuploid might participate in the immunosuppressive microenvironment. Hence, we analyzed the TMB, the immune score, the immune evasion situation and the infiltration of 28 types of immune cells between the aneuploid and the near-diploid ovarian cancer. Comparison analysis showed that there was no difference of TMB between the aneuploid and the near-diploid groups, although the TMB level in the aneuploid group seemed slightly lower than the near-diploid patients (Fig. 7A). The immune evasion analysis showed that the aneuploid ovarian cancers had a higher TIDE evasion score, mainly attributed to the exclusion instead of the dysfunction of T cells (Fig. 7B). Estimate score suggested that the near-diploid patients had higher immune score (Fig. 7C). Comparison analysis of diverse immune cell infiltration suggested that the activated macrophage, activated dendritic cell, effector memory CD8 + T cell, CD4 + T cell, activated CD8 + T cell, type 1 helper cell and type 17 helper cell were significantly lower in the aneuploid ovarian cancer than the near-diploid cancer (Fig. 7D).

**Fig. 8:**
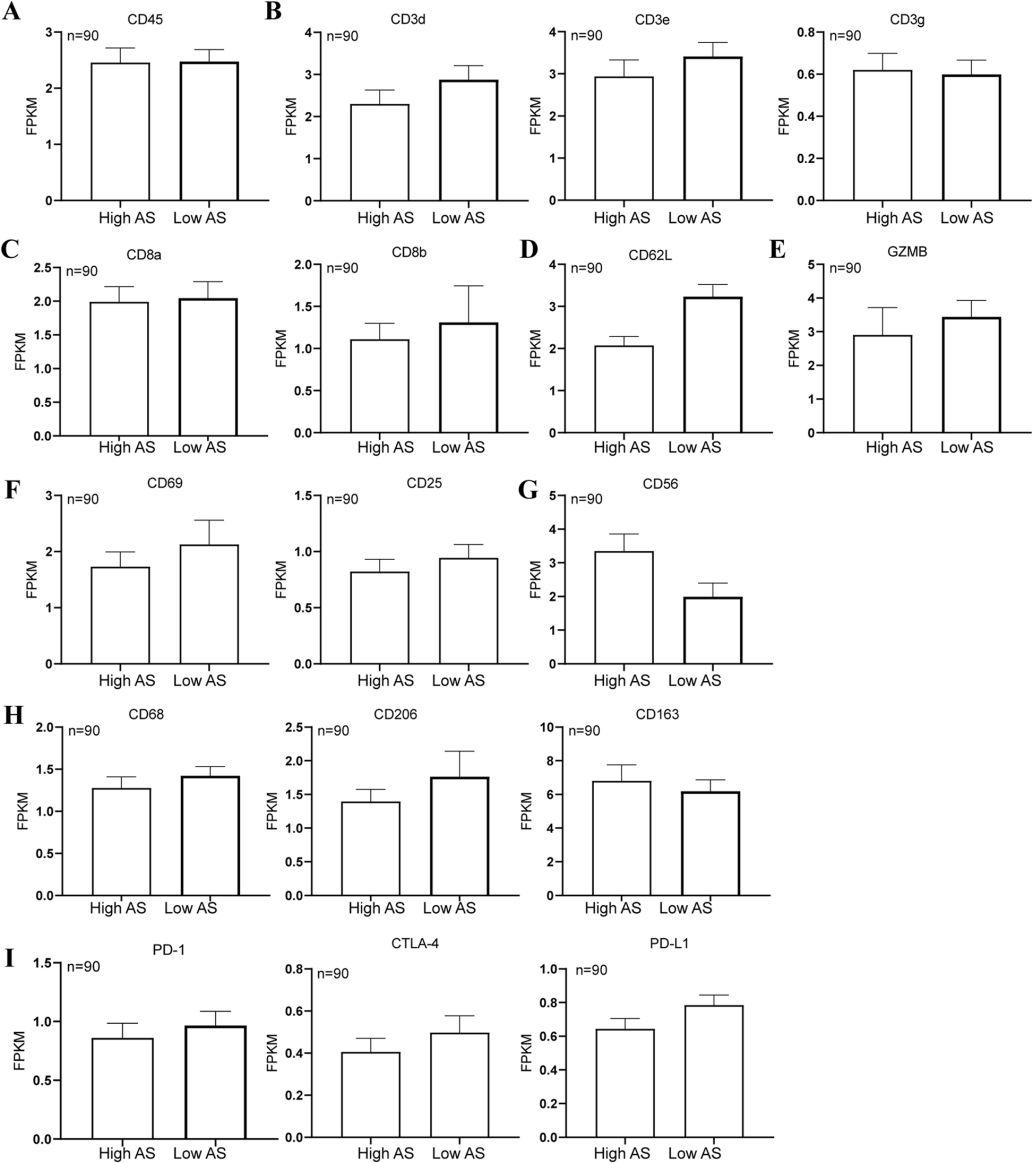
Comparison of immune infiltration and immune checkpoints between the highly-aneuploid and the near-diploid ovarian cancer patients. **A** Immune cell biomarker: CD45 (*P* > 0.05).** B** CD3 (*P* > 0.05). **C** CD8 biomarker (*P* > 0.05). **D** Biomarker of memory T cell: CD62L (*P* < 0.05). **E** Cytotoxic biomarker of T cell: GZMB (*P* > 0.05). **F** Activation biomarkers of T cell: CD69 (*P* > 0.05), CD25 (*P* > 0.05). **G** NK cell biomarker: CD56 (*P* < 0.05). **H** Macrophage biomarkers: CD68 (*P* > 0.05), CD206 (*P* > 0.05), CD163 (*P* > 0.05).** I** Immune checkpoints: PD-L1(*P* > 0.05), PD-1(*P* > 0.05), CTLA-4(*P* > 0.05)

**Fig. 9:**
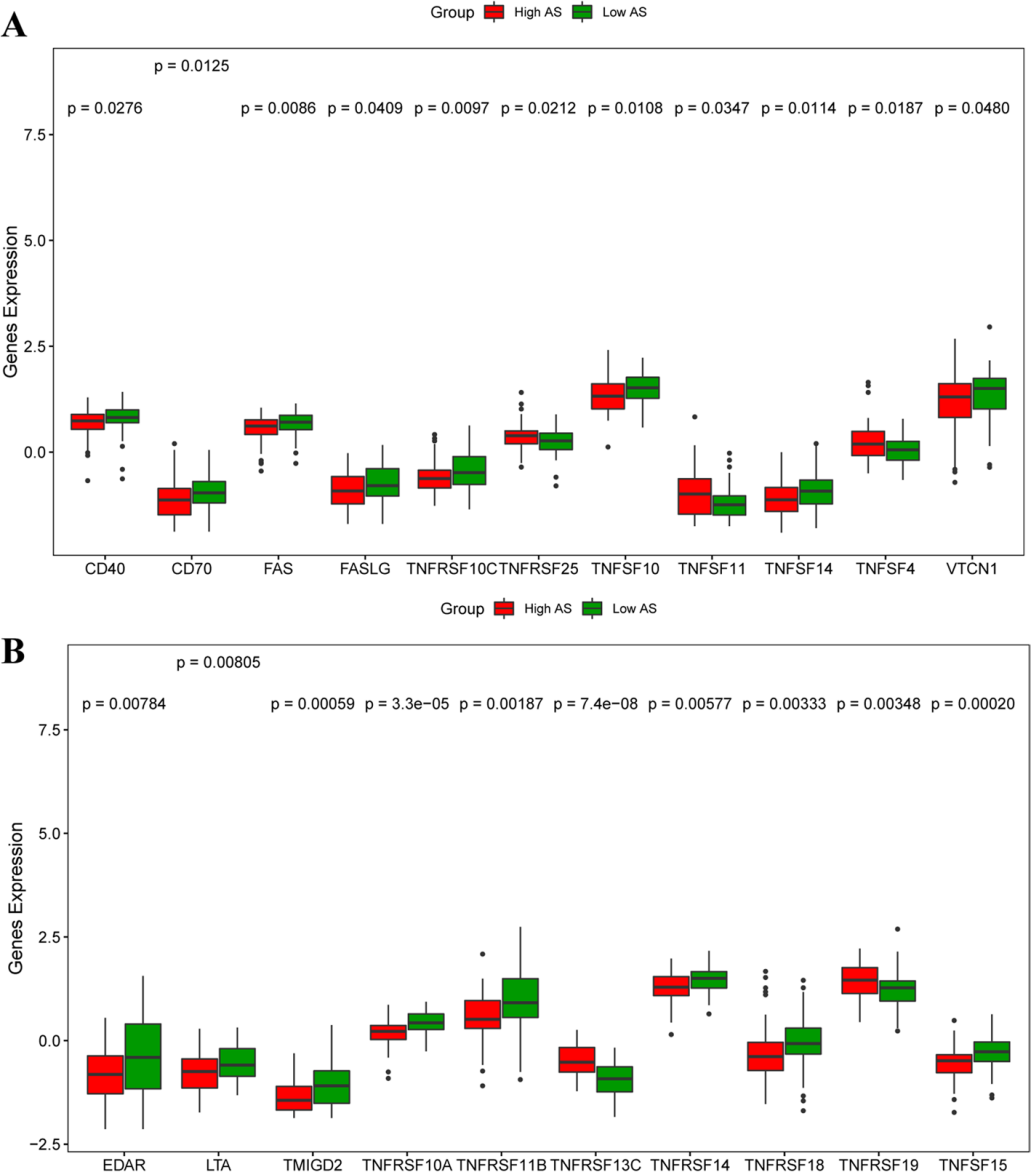
Comparison of immune checkpoints expression between the highly-aneuploid and the near-diploid ovarian cancer patients

Meanwhile, the immunohistochemistry data of immune markers in the HPA database showed that the level of CD62L (Fig. 8D), a biomarker of naive and memory T cell, was lower in the aneuploid ovarian cancer than the near-diploid group, while the level of CD56 (Fig. 8G), a biomarker of NK cell, was higher than the near-diploid patients. However, there was no significant difference of immune checkpoints between the two groups, including PD-L1, PD-1 and cytotoxic T lymphocyte antigen 4 (CTLA-4) (Fig. 8I). Furthermore, analysis of 60 immune checkpoint genes showed that the tumor necrosis factor receptor super family (TNFRSF) showed significantly different expression between the two groups, which might be a promising target for the aneuploid ovarian cancer (Fig. 9).

## Discussion

In this study, we first comprehensively analyzed the ploidy information of ovarian cancer combining the karyotype, ploidy value and aneuploid score of tumor tissues. According to the results from both the G-banding in Mitelman database and the low-pass whole genome sequencing in TCGA database, a high fraction of aneuploid was found in ovarian cancer. The ploidy-heterogeneity might provide significant clues for diagnosis and treatment of ovarian cancer.

Mutation might not be appropriately used for the precision management of ovarian cancer. Except for TP53 mutation, few other tumor suppressor or oncogene mutations existed in ovarian cancer, with less than 10% frequency in patients [15]. It was suggested that ovarian cancer might be categorized into “C” type cancer, dominated by copy number variations, instead of mutations [14, 23]. Our previous analysis showed that there was no difference between the aneuploid and the near-diploid patients in the frequency of the common mutations in serous ovarian cancer or the mutations highly appearing in any group [24]. These data indicated that ploidy and CNV level might be suitable for the identification of ovarian cancer subtypes, and therefore, might help the personalizing therapy tailored to each subtype.

Arm-level and whole chromosome alterations were also analyzed in our research. Ovarian cancer had a large scale of arm-level or whole chromosome changes. Overall, losses might occur more frequently than gains, regardless of arm-level or whole chromosome variation. For arm-level changes, CNV data showed that 17p loss, 8p loss, 16q loss and 22q loss are more common than other arm-alterations in ovarian cancer, consistent with previous cytogenetic data [25]. These data indicated the locus of other potential suppressor genes, except for TP53 on 17p, which might be useful for further anticancer exploration. Chr8 loss, chr15 loss, chr22 loss, chr 19 loss, chr X loss and chr 12 gain, chr 20 gain are common recurrent chromosome changes, which might partially drive the ovarian cancer initiation and progression. Comparison analysis suggested that the aneuploid subtype, instead of all ovarian cancer patients, mainly contributed to these common chromosome alterations in ovarian cancer.

The potential correlation between ploidy and clinicopathologic parameters supported the tumor-promoting role of aneuploid in ovarian cancer. It seems that the aberrant ploidy might play a dual role in cancer, tumor-inhibiting or tumor-promoting role, according to the information reflected by clinical samples and experiments [26]. However, overall, aneuploid and polyploid might be helpful for the evolutionary selection during cancer progression with their strong adaptive capacity [27]. In this study, aneuploid cancer correlates with higher grade and poor survival, consistent with previous cohort in ovarian cancer [13]. For further clarifying the role of the nondiploid in ovarian cancer, more experiments are needed using cell lines and paired ploidy models. Even so, the ploidy-clinical association in this study indicated that the aneuploid score, which can be calculated through low-pass whole genome sequencing and ABSOLUTE algorithm, might be used as a convenient biomarker for diagnosis or precision of ovarian cancer [16].

In this study, we found the ploidy-MHC association and first analyzed the correlation between aneuploid and the infiltration of multiple immune cells on the basis of expression and protein level in ovarian cancer. Immunotype classification showed that most ovarian cancers belonged to T cell “exclusion” phenotype or T cell “cold” phenotype [28]. Our research suggested that the highly-aneuploid might contribute to the immune “desert” in ovarian cancer. Aneuploid correlated with abnormal expression of MHC I and MHC II class, which indicated an abnormal capacity of antigen processing and presentation in the nondiploid ovarian cancer. Transcription and protein data showed that there was less infiltration of macrophage, activated dendritic cell, activated CD8 + T cell, central memory T cell in the aneuploid than the near-diploid ovarian cancer. Meanwhile, the TIDE analysis showed that T cell exclusion was significantly more frequent in the aneuploid than in the near-diploid. This supported that nondiploid cancer cells might promote immune evasion via low HLA abundance, and the exclusion and dysfunction of T cells in ovarian cancer. The ploidy-immune association might provide clues for mechanisms of immunosuppressive microenvironment in ovarian cancer, although the underlying crosstalk between nondiploid cancer cells and immune cells still remains to be clarified.

The potential ploidy-immune relationship also provided some inspirations for ovarian cancer immunotherapy. Recently, a phase I clinical trial of patients with non-small cell lung cancer suggested that the concurrent radiotherapy and ICB showed better efficacy than sequential regimen, depending on the existing highly-aneuploid cancer cells before treatment [29]. Surprisingly, radiotherapy alone decreased fraction and function of T cells and adaptive immune. But the concurrent regimen might realize immune activation. This reminds us of the genetic vulnerability of the highly-aneuploid cancer cells [27].

Radiation therapy causes DNA damage and plays a therapeutic role through breaking DNA of cancer cells [30]. Similarly, the poly ADP-ribose polymerase inhibitor (PARP inhibitor) realized anticancer efficacy through synthetic lethality in patients with BRCA1 or BRCA2 mutation [31]. The synthetic lethality was thought to be caused by both the existing disability of DNA damage repair in cancers with BRCA mutation and the induced DNA damage by PARP inhibitor. The broken DNA from PARP inhibitor and radiation might trigger the immunogenic cell death through increasing the neoantigen and activating T cell and NK cell via cGAS-STING signaling [32]. However, recent ploidy research in ovarian cancer might enlarge the population of PARP inhibitor [33]. This research suggested that aneuploid showed sensitivity on PARP due to its existing genome instability, similar to synthetic lethality by PARP and BRCA mutation. Combining the superior PARP response and the increased ICB efficacy in the aneuploid than the near-diploid, aneuploid cancer cells might provide potential neoantigen for immune activation and cancer therapy.

The largest limitation of this study is that the interaction between aneuploid cancer cells and immune cells was not verified via experiments. Despite this limitation, the ploidy information and ploidy-immune analysis in this study still provide inspirations for immune activation and cancer therapy in ovarian cancer.

## Conclusions

In conclusion, we first comprehensively analyzed the ploidy information and recurrent arm-level or whole chromosome changes in ovarian cancer combining the karyotype and sequence data. Differentially expressed genes between the near-diploid and the aneuploid cancers were identified and analyzed. Upregulated genes in aneuploid patients were enriched in collagen-related signaling, whereas decreased genes were involved in immune function. Furthermore, the ploidy-immune relationship was explored and verified through enrichment analysis and the Human Protein Atlas database.

Overall, aneuploid was widespread and prominent in ovarian cancer, which might be used for survival prediction and precision management. The ploidy-immune association indicated that aneuploid might shape the immunosuppressive microenvironment in ovarian cancer. Future works need to focus on the interaction between nondiploid cancer cells and immune cells. Moreover, the ploidy-related vulnerability might promote the anticancer therapy development and immune activation, which remains to be explored in experimental studies.

## Data Availability

Ploidy data and aneuploid score can be obtained from the cBioPortal website (www.cbioportal.org). Karyotype data can be obtained from the Mitelman database (https://mitelmandatabase.isb-cgc.org/).
